# New approaches to investigating social gestures in autism spectrum disorder

**DOI:** 10.1186/1866-1955-4-14

**Published:** 2012-05-24

**Authors:** Kenneth T Kishida, Jian Li, Justin Schwind, Pendleton Read Montague

**Affiliations:** 1 Human Neuroimaging Laboratory and Computational Psychiatry Unit, Virginia Tech Carilion Research Institute, Roanoke, VA, 24016, USA; 2 Department of Psychology, New York University, New York, NY, 10003, USA; 3 Department of Athletics, University of South Alabama, Mobile, AL, 36608, USA; 4 Department of Physics, Virginia Tech, Blacksburg, VA, 24061, USA; 5 Wellcome Trust Centre for Neuroimaging, 12 Queen Square, London, WC1N 3BG, UK; 6 Virginia Tech Carilion Research Institute & Department of Physics, Virginia Tech, 2 Riverside Circle, Roanoke, VA, 24018, USA

**Keywords:** Autism spectrum disorder, Social exchange, Reciprocation, Game theory, Computational models, Functional magnetic resonance imaging, Biomarker

## Abstract

The combination of economic games and human neuroimaging presents the possibility of using economic probes to identify biomarkers for quantitative features of healthy and diseased cognition. These probes span a range of important cognitive functions, but one new use is in the domain of reciprocating social exchange with other humans - a capacity perturbed in a number of psychopathologies. We summarize the use of a reciprocating exchange game to elicit neural and behavioral signatures for subjects diagnosed with autism spectrum disorder (ASD). Furthermore, we outline early efforts to capture features of social exchange in computational models and use these to identify quantitative behavioral differences between subjects with ASD and matched controls. Lastly, we summarize a number of subsequent studies inspired by the modeling results, which suggest new neural and behavioral signatures that could be used to characterize subtle deficits in information processing during interactions with other humans.

## Review

The challenges of social exchange are shared throughout the animal kingdom [1-3]. Humans engage in repeated reciprocal interactions with kin and non-kin; this has required our species to develop a particular capacity to track these interactions and assign credit and blame accordingly [3-6]. The underlying neurobiological mechanisms underlying these abilities in humans remain an open area of investigation, which is generating insight into a number of mental illnesses including autism spectrum disorders (ASD) [7-18].

One important challenge for an agent engaged in social exchange is the ability to generate models of others’ mental states, a capacity referred to as ‘theory of mind’ [19,20]. Human neuroimaging experiments have implicated a consistent set of brain regions hypothesized to be involved in this process [21-24]. The role of this kind of computation and others are exemplified in even the simplest exchanges. For instance, in a fair trade the brains of the interacting agents must be able to: (1) compute norms for what is considered fair; (2) detect deviations from such norms; and (3) select appropriate actions based on these deviations.

Reciprocating social exchanges and their related computations occur in our daily lives and are the basis for a number of staged interactions in clinical practice. However, the computations themselves likely occur well below our threshold for conscious experience. For example, in the diagnostic procedures for ASD a trained professional interacts with a suspected patient and navigates a predetermined series of give-and-take scenarios (for example, the Autism Diagnostic Observation Scale (ADOS) [25]); during this interaction the clinician tries to detect typical and atypical social gestures based on trained-up (for example, experience-based) expectations of behavior. Additionally, insight into the patient’s social cognitive process is sought using interviews of the patient and family members [26,27].

Computational approaches in combination with quantitative probes of behavior (for example, game theoretic paradigms [28,29]) promise to reveal underlying dimensions of healthy human decision-making and thereby will provide a basis for which to compare against in psychiatric populations [30,31]. These approaches promise to excel, over traditional approaches, in capturing quantitative normative behavior and associated neurobiological responses. In these tasks participants are required to make decisions under incentivized conditions; the incentive is typically a monetary one and the decision spaces are restricted by the particular game [28]. The games may be against a ‘roll of the dice’ or against real or simulated agents (for example, other humans or computer simulations) and can be employed though a computer interface. These conditions can augment traditional diagnostic procedures by providing additional behavioral and neurobiological measurements. In computer-based game play, the behaviors elicited and signals exchanged between players are all captured within the patient-computer interface. These measurements can then be directly ported into tests over competing computational models about hypothesized cognitive processes underlying social behavior. In addition, variables within the games are designed to be parametric, which provides increased specificity in the analysis of associated brain responses. The use of the internet and technologies like hyperscanning, which synchronizes fMRI scanners and study participants over the internet [32], has already and will continue to allow more complex social exchange to be studied while allowing experimenters to have control over which signals are exchanged. These methods take highly complex social interactions and reduce the exchanges to simpler, more tractable, dimensions for quantitative analysis; and, have recently been applied to the neurobiological investigation of social exchange [33-38]. Fairness and cooperation games dominate these recent efforts because they assess a subject’s internal norm for what is fair in an exchange, and they require that each subject model their partner’s mental state [28,39-45]. These games are excellent experimental probes because they are simple, there is an existing body of behavioral data employing them across a variety of contexts, and there are known solution concepts for how they ‘should’ be played by a rational self-interested agent [28,40,41]. Importantly, these games all require participants to model their partner. Such simplified behavioral probes provide a starting point for extracting quantitative descriptions of social signaling and its pathologies. These games are beginning to prove valuable in early work in clinical populations such as borderline personality disorder [34] and ASD [45,46].

### Games to study autism spectrum disorders

**Figure 1:**
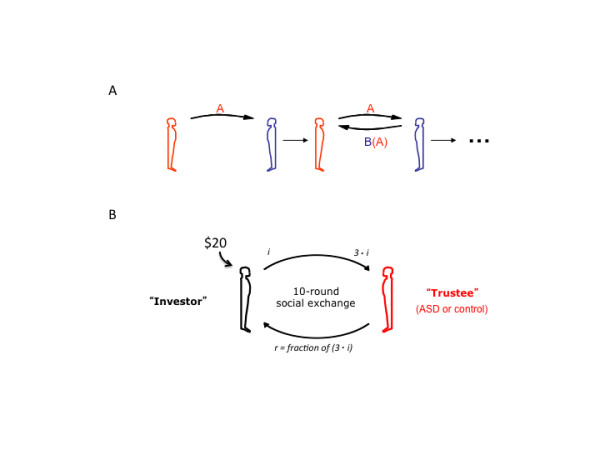
**Two-party social exchange games to probe autism spectrum disorder neurobehavioral responses.** (**A**) Two-party repeated interaction games with feedback and learning. Two-party signaling games allow for the investigation of social exchange between two (or more) agents. The signals sent between participants are controlled such that the information sent and the information that must be inferred is explicit and controlled by the experimenter. Multi-round games allow for the development of reputation and the opportunity for learning and adapting, which allow for the study of interesting social dynamics in a controlled and objectively quantitative manner. (**B**) Multi-round trust game. The multi-round trust game is a 10-round repeated interaction between the same two partners. Player 1 (‘investor’) is endowed with 20 points and is to decide how much, if any, to share with the ‘trustee’ (player 2); the amount shared is tripled on its way to the trustee; the trustee then decides how much, if any, to repay to the investor; at the end of each round the total points earned/kept by each player is put into a ‘bank’; and the subsequent round begins with a new endowment of 20 points.

The economic games employed in human decision-making experiments range from relatively simple gambling games to more complex social signaling tasks [40]. The latter has recently been employed to study social exchange and associated mentalizing operations in participants diagnosed with ASD [45,46]. These studies used two-person sequentially iterated games with multiple rounds (Figure 1A). These games are mathematically more complicated than one-shot exchange games found in the economic literature, but they allow the observation of iterated exchange where signaling between participants can provide valuable learning and guidance cues that are analogous to the kinds of signals we use during more natural social exchange. The two earliest games to be used to probe social behavior in an ASD cohort include the multi-round trust game (Figure 1B[46]) and the stag-hunt game [45]. Both of these games possess an optimal strategy where the players cooperate; however, participants must infer their partner’s strategy from the behavioral signals exchanged within the context of the game and if they are wrong they may incur costly penalties for misjudging the cooperativeness of their partner. The reduction of the space from where social signals originate in these games (compared to more naturalistic settings) provides an ability to explicitly differentiate what signals are being exchanged from those that must be inferred. This reduction of the behavioral space is a benefit for the application of computational approaches to the behavioral data and has begun to reveal interesting quantitative aspects required for healthy social exchange. The multi-round trust game (Figure 1B) and the stag-hunt game are played between two agents with repeated interactions. This allows participants to learn from signals sent and the feedback received in subsequent stages of the game (Figure 1A). These games have been described in detail elsewhere including the respective theoretical development of the notions of optimal play, however for the purpose of discussion we will briefly describe the main rules for each game below.

The multi-round trust game (Figure 1B, [34,46,47]): (1) there are two players, an anonymous ‘investor’ and an anonymous ‘trustee’; both players have full knowledge of what the other player’s options are given the rules of the game; (2) the investor is given 20 points to invest with the trustee (should they decide to); (3) once an investment is made (range of possibilities = 0 to 20 points in 1-point increments) the trustee receives three times the investment and the investor keeps what they did not invest; (4) now the trustee decides how much to reciprocate to the investor (the range of possibilities = 0 to 3 times the investment, in 1-point increments); (5) the trustee keeps what they did not reciprocate and the amount reciprocated is added to the investor’s total; (6) at the end of each round the totals for each player are shown to both players and ‘deposited’ into each of their respective banks; (7) this game is repeated for a total of 10 rounds where each round starts with a fresh 20 points and the totals at the end of each round are put aside to be tallied up at the very end of the game; (8) at the end of 10 rounds the totals in the bank for each player determines how much real money they will be paid and is how the game is incentivized: the more points one earns the more real money they will take home. To earn the most points possible the players must cooperate; however, generous signals of cooperation can be taken advantage of and a player may cheat the other out of an equal distribution of the profits.

The stag-hunt game also engages two participants and also requires participants to cooperate to achieve maximal gains. The goal of the game is to ‘hunt’ either a ‘stag’ (big profits) or a ‘rabbit’ (smaller profits). The players see a game board where they can move one square at a time and do so sequentially. In this manner, the participants observe the behavior of their partner and attempt to infer whether the move was intended to hunt the stag (cooperate) or a rabbit (defect). The rabbits do not move and are positioned on the board such that they can be hunted by the efforts of a single player, but provide a small payoff; on the other hand, two players must cooperate to hunt the stag and are rewarded for doing so with a bigger point value. Each move in the game that does not collect either a rabbit or a stag is viewed as a cost and diminishes a player’s point total. In the implementation of this game by Yoshida and colleagues the stag moved first, then the human participant, then a computer agent partner [45]. Like the multi-round trust game the players point totals are directly tied to the amount of real money they will take home at the end of the experiment, thus incentivizing participants to make truthful expressions of their preferences and assessments of the game state.

Yoshida and colleagues’ results from applying the stag-hunt game to investigate hidden cognitive processes such as belief inference in autistic populations is particularly promising for our understanding of ASD and for computational and game theoretic approaches to investigating the heterogeneity known to exist within the spectrum [45]. The application of their ‘game theory of mind’ [43] demonstrated an ability to differentiate ASD symptom severity by a parameter in their model related to the patient’s ability to infer the strategic sophistication of their partner; the authors clarify this as the ability to infer the partner’s mindreading strategy. Additionally this parameter was distinct from the models estimate of patients’ ability to plan iteratively, which was not related to symptom severity, but rather showed a strong correlation with intellectual ability as assessed by verbal and non-verbal intelligence tests [45].

### Early results from the multi-round trust game

The multi-round trust game has been used to investigate social exchange in pairs of healthy individuals [34,46-48] and pairs consisting of a healthy investor and a trustee diagnosed with a psychiatric disorder including: ASD [46,48], borderline personality disorder [48,49], major depression [48], and attention deficit hyperactivity disorder [48]. Tomlin and colleagues identified a specific response pattern along the anterior to posterior axis of the cingulate cortex [47]. They identified that a spatial pattern of activity that corresponded with ‘self’ and ‘other’ phases of the trust game exchange. This agent-specific response did not modulate with the number of points exchanged, the character of the gesture (benevolent or malevolent [34,47]), or role (investor or trustee) of the player; the response pattern was specific to who was acting at that particular stage of the game (that is, ‘me’ or ‘not me’). Additionally, the agent-specific pattern of activity was absent when the players participated in a control experiment where the partner was absent and the players knowingly engaged in a computer-driven task [47]. This result suggested that the cingulate ‘self’ and ‘other’ response patterns may be affected in special populations where these signals would be important for social exchange.

**Figure 2:**
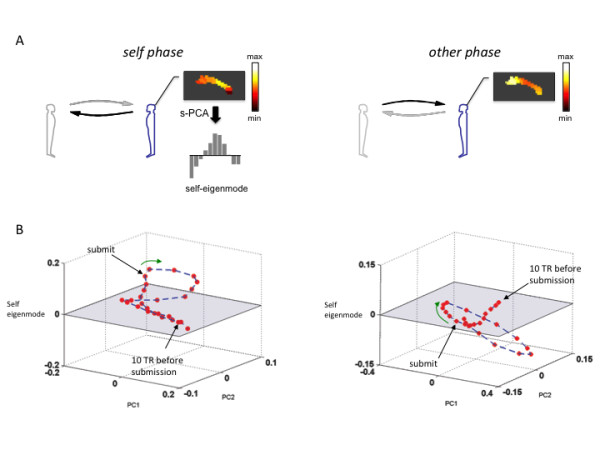
**Spatial principal components analysis identifies ‘self-eigenmode’ response during social exchange (adapted from [**[46]**]).** (**A**) Cingulate hemodynamic responses from the trustees’ brain during the ‘self’ and ‘other’ phases of the multi-round trust game. The spatially defined domains along the posterior to anterior axis were subjected to principal components analysis. Among the principal components identified was the ‘self-eigenmode’, which captures the dynamic agent-specific spatio-temporal activity in the cingulate as the trust game evolves. The self-eigenmode flips its sign as the game transitions from the self-phase to the other phase of the trust game. (**B**) Phase dynamics of cingulate response during multi-round trust game. The self-eigenmode response does not evolve in isolation. Three eigenvectors characterize the full set of the cingulate self-basis. Each of the red circles is a single TR in the measurement of the BOLD response during the self and other phases of the trust game in the trustee brain. These responses are plotted in the three dimensions that comprise the cingulate self-basis and shows how the cingulate response is dynamic, yet regular and throughout repeated trials of the self and other phases of the trust game. Increased self-responses are indicated by more positive values on the self-eigenmode axis (vertical axes); whereas non-self or ‘other’ responses are indicated by more negative values on the self-eigenmode. On the left we observe positive values on the self-eigenmode dimension leading up to an following the submission of a repayment by the trustee; and, on the right we observe negative self-eigenmode values in the trustee brain following the submission of an investment by the investor.

**Figure 3:**
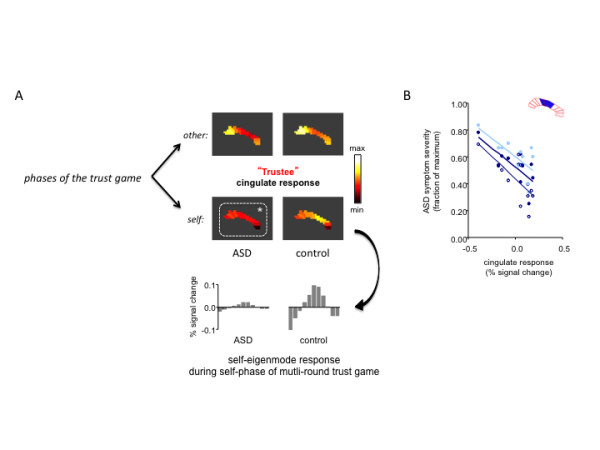
**ASD trustees show diminished cingulate self-response pattern during social exchange in the multi-round trust game (adapted from [**[46]**]).** (**A**) Diminished cingulate response pattern during ‘self–phase’ of the iterated multi-round trust game. The ‘other’ response pattern was similar in ASD (*n* = 12) and age- and IQ-matched control participants (*n* = 18). However the cingulate ‘self’ response was diminished in the ASD cohort. Projection of the BOLD response pattern onto the cingulate self-eigenmode reveals the contribution of that mode of operation in the cingulate, and show that it is significantly diminished in the ASD cingulate cortex. (**B**) Regions in the middle cingulate cortex show diminished response, which correlates with ASD symptom severity. The middle regions of the cingulate cortex show significant correlation with symptom severity in the ASD cohort. The less active this region is during the self-phase is correlated with the communication subscale, social subscale, and overall score on the Autism Diagnostic Interview, Revised (open circles: ADI-R communication subscale, r = −0.69, *p* = −0.012; light blue filled circles: ADI-R social subscale, r = −0.70, *p* = 0.011; dark blue filled circles: ADI-R total score, r = −0.73, *p* = 0.007).

Chiu and colleagues tested this hypothesis by investigating social pairs consisting of a healthy investor and a trustee diagnosed with ASD [46]. This work shed light on the role of the agent-specific cingulate response patterns during social exchange and on altered neural responses in patients diagnosed with ASD. The authors used an analytical approach to disentangle various modes of operation in the cingulate cortex using principal components analysis applied to spatio-temporal data measured in the cingulate during the social exchange game. One mode they identified matched the previously observed agent-specific response profile and was named the ‘self-eigenmode’ due to the specific phase of social exchange in which it was elicited (Figure 2). Determining the self-eigenmode allows the reduction of the spatial pattern of activity to a single dimension and compares the role this ‘response pattern’ plays in social exchange iterations. Chiu and colleagues went on to show that this pattern was also elicited during perspective taking shifts in a structured imagery task [46] and demonstrated that participants with ASD showed diminished responses along the self-eigenmode specifically during the self-phase of the trust game (Figure 3A[46]). These results led to the demonstration that a region in the middle cingulate cortex (the peak regions in the self-eigenmode) showed diminished activity in the ASD participants that correlated with their symptom scores on the Autism Diagnostic Interview, Revised [26] social exchange and communication sub-scales, but not the repetitive behaviors scores; the diminished cingulate self-response also correlated with their overall ADI-R score (Figure 3B[46]).

**Figure 4:**
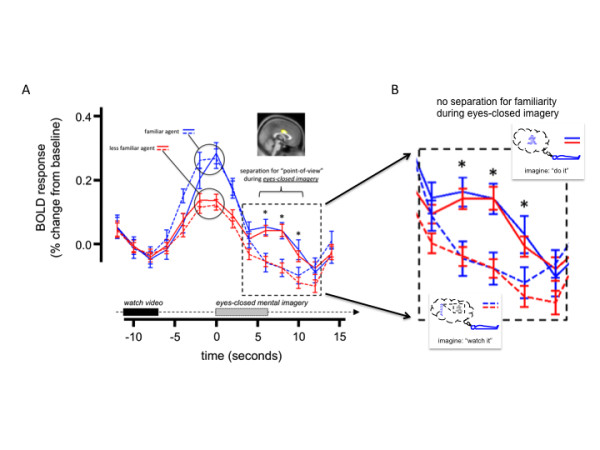
**Middle cingulate cortex differentiates perspective taking responses during eyes-closed imagery.** (**A**) Time-series of BOLD response in middle cingulate cortex during structured imagery task. (Inset: mask used in time-series analysis.) Mean BOLD response (% signal change from baseline) along the vertical axis; time (s) along the horizontal axis. Plotted: mean response of the middle cingulate cortex (MCC) ± standard error about the mean for motor-imagery trials (solid lines) and visual-imagery trials (dashed lines) for expertise-congruent conditions (blue lines) and expertise-incongruent trials (red lines). Note: the MCC responds during motor-imagery trials, but not during visual-imagery trials (solid lines *vs.* dashed lines, time-points +4 s to +12 s, see dashed box), and the MCC does not differentiate expertise-congruent trials from expertise-incongruent trials during eyes-closed visual or motor imagery (blue lines *vs.* red lines, time-points +4 s to +12 s). Asterisks: *p* < 0.05, (one-tailed t-test, *n* = 81 subjects: ‘do it’ > ‘watch it’ collapsed over congruency conditions). (**B**) Middle cingulate response during eyes-closed imagery. Familiarity with subject and action (for example, athletic expertise congruency) does not separate MCC response during eyes-closed imagery; however, first-person perspective-taking does.

**Figure 5:**
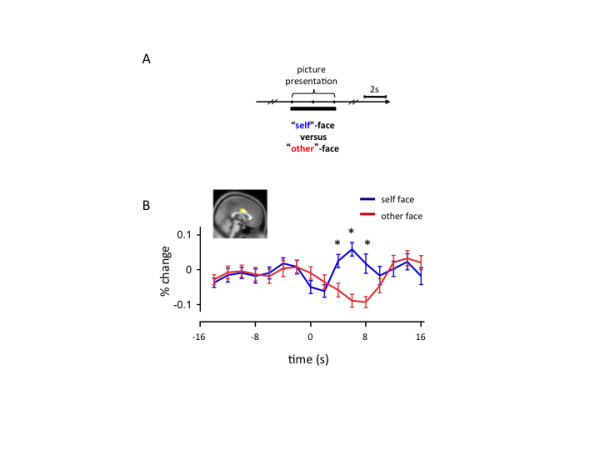
**Self and Other Faces task shows visual response in middle cingulate cortex.** (**A**) Self-Other Faces task paradigm. Twenty-five healthy adult participants were shown pictures of themselves (15 unique pictures, eyes forward and smiling) and pictures of another gender-matched volunteer (15 unique pictures, eyes forward and smiling). The pictures were shown for 4 s and in a randomized order. The inter-trial interval was jittered using 2 s, 4 s, or 6 s. (**B**) Time-series of BOLD response in middle cingulate cortex during Self-Other Faces task. (Inset: ROI mask derived from structured imagery task (see Figure 4)). Mean BOLD response (% signal change from baseline) along the vertical axis; time (s) along the horizontal axis. Pictures are displayed at time = 0 s for a duration of 4 s. Error bars: Standard error around the mean; asterisks: *p* < 0.05, (one-tailed t-test, *n* = 25 subjects: ‘self’-picture response > ‘other’-picture response).

We hypothesize that perspective-taking responses may be elicited automatically during the first moments of engaging another agent preceding active social exchange. To investigate brain responses consistent with this hypothesis we looked for responses in the athletes’ brains (during the structured mental imagery task [46]) that were active during eyes-closed perspective-taking and during visual observation of athletically active agents. Additional file 1: Figure S1 shows the paradigm executed by the accomplished athletes (adapted from [46]). Also, in Additional file 2: Figure S2, we show that the self-response is greatly diminished when individuals perform eyes-closed visual imagery (a kind of third-person perspective-taking), which is comparable to the response observed in the ASD participants when they ought to be showing a characteristic cingulate self-response (Figure 3A). A conjunction analysis (*n* = 81, *p* < 0.001, FDR corrected) looking for regions active in response to eyes-closed first-person perspective-taking and self-congruent visual stimuli identifies the middle cingulate cortex (inset Figure 4A), bilateral caudate, and the thalamus (not shown). Among these regions, the middle cingulate cortex (inset Figure 4A) is unique in the time series of the BOLD response (Figure 4A). Consistent with the GLM contrast, the middle cingulate cortex differentiates first-person from third-person perspective-taking, and self-congruent from self-incongruent visual stimuli (video); however, unlike the other regions identified in this contrast, we show that the middle cingulate cortex does not demonstrate a change in response to self-(in)congruency (that is, familiarity) during the eyes-closed mental imagery phase of the task (Figure 4A dotted box and expanded in Figure 4B). This result suggests that the diminished middle cingulate responses observed in the ASD participants may be due to diminished first-person perspective-taking ability, which is consistent with criteria described in the DSM-IV-TR. The fact that the video clips elicit responses in the middle cingulate cortex that do differentiate degrees of familiarity suggest it may be feasible to test responses in this region using simpler ‘self’ and ‘other’ stimuli. We tested this hypothesis using a simple self and other picture task (Figure 5). Twenty-five healthy adult participants viewed pictures of themselves and another person while undergoing an fMRI brain scan (Figure 5A shows faces task paradigm). We performed a region of interest inspection of the time-series of the BOLD response in the middle cingulate cortex using a mask (Figure 4A inset) derived in the structured imagery task ( Additional file 1: Figure S1). Figure 5B demonstrates visual responses in the middle cingulate cortex that differentiate pictures of ‘self’ from pictures of an ‘other’. This kind of simple task may be used to identify deficient self-responses in individuals diagnosed with ASD. Note: these analyses were carried out on data collected with the approval of the Institutional Review Board at Baylor College of Medicine under guidelines in compliance with the Helsinki Declaration.

### Extracting useful models during staged social exchanges

The multi-round trust game is a relatively rich task between two interacting subjects that requires several cognitive capacities to be intact for normal patterns of exchange. Ultimately, models of such exchanges will have to take account of the load they impose on memory systems, valuation systems, the capacity to recall and adjust norms for fairness, the capacity to model one’s partner, and so on. Here, we describe some first attempts to capture an important aspect of this reciprocating exchange in a principled computational model [50]. The idea is based directly on the natural signal class in this game: deviations from fair exchanges, or to be more particular, deviations from neutral reciprocity. In this task, neutral reciprocity is equivalent to ‘tit-for-tat’ behavior where one subject matches (on average) the fractional changes in money sent across rounds by their partner. Deviation from such an exchange is known to be the main ‘signal’ that causes one’s partner to change their behavior [34]. The prevailing utility model for this game is the inequality aversion model of Fehr and Schmidt [51], which also provides a natural way to ‘type’ players. Player i’s valuation of their immediate payoff is defined as:

(1) $$U_{i}(x_{i},x_{j};\alpha_{i},\beta_{i})=x_{i}-\alpha_{i}max(x_{j}-x_{i},0)-\beta_{i}max(x_{i}-x_{j},0)$$

Where *x*_*i*_ is the amount of money acquired by player i and similarly for player j. The *α*_*i*_ and *β*_*i*_ parameters model envy (player j gets more than player i) and guilt (player i gets more than player j). In Ray *et al*. [50], these parameters were taken as the ‘type’ of each player in the exchange and hence stratifies types of players according to their reaction to inequitable exchanges. That model used the observed patterns of monetary exchange to infer the type of each player and also included an estimation of the most likely depth-of-thought used by each player in the exchange - that is, how deep into the interaction each player simulated in order to decide on their next move. This is a potentially important estimate in the context of ASD since this kind of parameter may provide a new way to characterize one part of afflicted individual’s differences in socio-emotional reciprocity. The model provided a full generative model that was able to capture broad classes of behavior observed in subjects carrying out the trust game, but it has yet to be applied directly to subjects with ASD playing the game although these data exist [46].

**Figure 6:**
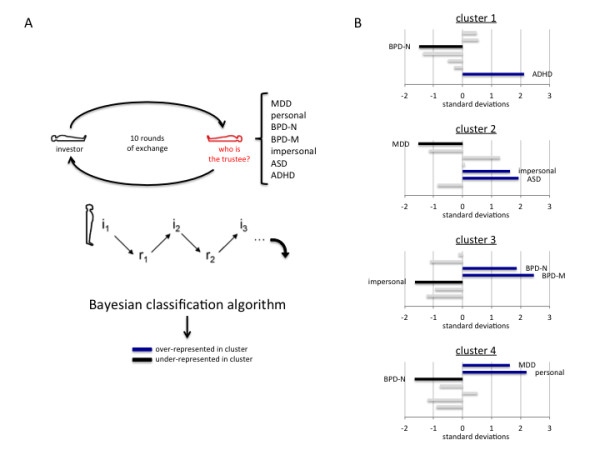
**Classification of Trustee ‘type’ from Investors’ behavior in two-party exchange.** (**AB**) Depiction of model-free clustering approach using multi-round trust game data. The data used in this approach was collected in previous studies [34,46-49] A. The multi round trust game is played between a healthy investor (black player) and a ‘target’ trustee (red player). The ‘target’ trustee was one of the following ‘types’: major depressive disorder (MDD), personal [47], borderline personality disorder - non-medicated (BPD-N) [49], borderline personality disorder - medicated (BPD-M) [49], impersonal [34], autism spectrum disorder (ASD) [46], and attention deficit hyperactivity disorder (ADHD) [47]. The approach described in detail in Koshelev *et al*. (2010) examines the investor behavior as a polynomial of past rounds of investments and returns (see panel B). i_1_, i_2_, … , i_t_ are the investments made by the investor during round t. Likewise, r_1_, r_2_, … , r_t_ are the repayments made by the trustee during round t. *Classification of the investor-trustee dyad*: is performed by predicting the investors’ decision at round t using a polynomial where the order of the polynomial, the number of past rounds, and the number of clusters discovered are left as free parameters to be discovered. The diagnostic categories for the trustee ‘type’ listed in panel A are blinded in this classification procedure. Only the behavior (investments and repayments over rounds) in the multi-round trust game is used. The result of this classification determined that a first-order polynomial, two rounds back, and four clusters were optimal. (B) Resulting four clusters and classification of diagnostic categories for model-free biosensor approach. The resulting classification identified four clusters where individuals from each diagnostic category were over- (blue bars) or under-represented (black bars). Vertical axes: investor-trustee dyad types (from top to bottom): MDD, personal [47], BPD-N [49], BPD-M [49], impersonal [34], ASD [46], and ADHD [47]. Only the statistically significant categories (that is, greater than 1.5 standard deviations from the mean) are labeled in each plot. Horizontal axes: standard deviations from the mean. Figure adapted from [48].

A second model-based approach to social exchange in subjects with ASD has been proposed by Koshelev and colleagues (Figure 6[48]). The approach used by Koshelev *et al*. is computationally involved, but the idea behind it is rather straightforward. Objectively measured behavioral signals (iterated investments and repayments) can be used to classify pair ‘types’, for instance healthy pairs *vs.* pairs consisting of a healthy investor and a patient (from a number of different psychopathologies (Figure 6A)). The approach takes advantage of the possibility of subtleties in game play that will allow the differentiation of patient populations (for example, participants diagnosed with ASD play differently than participants diagnosed with BPD). Interestingly, the differences in pair types are read out by looking at differences in how the healthy investor responds to the various patient populations in the trustee role; this highlights a biosensor approach as described in their report [48]. Briefly, Koshelev and colleagues used a Bayesian classification algorithm to agnostically classify the observed relationship between investments and repayments in preceding rounds and the next investment (Figure 6A). The investor’s decision at round ‘t’ (i_t_) is predicted using a polynomial, which incorporates previous investment (i_t-n_) and repayment (r_t-n_) decisions by both partners (Figure 6A).

(2) $$i_{t}=\beta_{0}\text{ + }\beta_{1}i_{t-1}\text{ + }\beta_{2}r_{t-1}\text{ + }\beta_{3}i_{t-2}\text{ + }\beta_{4}r_{t-2}$$

The number of rounds back (‘t-n’), the order of the polynomial, and the number of clusters to be identified were all left as free parameters to be discovered in their approach. Here, a Bayesian classification algorithm is applied to the regression coefficients (β). One important feature of this approach is that it focuses on how the history of the game impacts the decisions of the healthy investors. In all cases where psychopathology groups were used as subjects, these players were always in the trustee role (Figure 6A). Hence, studying the impact of such groups on investor behavior uses the investor brain as a kind of biosensor for features of the traditional psychopathology group behavior as expressed through this reduced two-person exchange. Figure 6B shows the outcome of using this method on a very large population of dyads where a healthy investor played a trustee drawn from a number of psychopathology groups. The approach is different from traditional cluster analysis in that a probability distribution is estimated for all clusters so that ASD subjects have a probability measure of being in each cluster. ASD subjects were over-represented in cluster 2 as shown in Figure 6B.

Work on these and similar models is underway and will require large amounts of normative data to be able to develop individual differences metrics useful in the real-world of clinical applications. So while these results are very preliminary, they are promising.

### Discussion and future directions

Social impairments are among the defining characteristics of ASD (from DSM IV-TR) [52]. Several influential theories have been posited to explain these social impairments seen in autism. The most prominent hypotheses implicate anomalies in theory-of-mind, joint attention, and functional cortical organization that contribute to social deficits in ASD. Together, studies across these domains highlight that individuals with autism perform quite well in some tasks that require inferring the beliefs and intentions of others, yet show marked deficits in other social inferences. The current state of the art in ASD research suggests there is significant value for probing ASD subjects using staged social exchange games paired with modeling approaches for estimating objective parameters associated with deficits in social computations.

To the authors’ knowledge, Yoshida and colleagues are the first to report a computational model of theory-of-mind processing applied to an ASD cohort [45]. In this work they take advantage of the staged interaction of the stag-hunt game. Like the trust game the stag-hunt game incentivizes cooperation, but players must infer from the behavior emitted from their partner whether cooperation is being reciprocated and whether it remains the most efficacious strategy. If the players infer a defection from cooperation then the behavioral strategy must be flexibly adapted. To develop their ‘game theory of mind’ [43] the authors represent the goals of each player with value functions derived from the machine learning literature. Given two players in the stag-hunt game the authors develop an estimation for their joint value function and recursively iterate: my representation of your value functions, your representation of my value functions, my representation of your representation of my value functions, and so on. The authors assume that this recursion is bounded; in other words, that it does not not go on *ad infinitum* and they estimate the depth of thought (that is, sophistication) from the expressed behavior of the players. Importantly, this mathematically expressed theory-of-mind is only one form of the kind of computational expression that may capture the kinds of inferences that occur during processes such as mentalizing or that may reveal aberrant computations carried out in the behavior and neural processes in individuals with autism. Other models have been developed [50,53,54], but have not been applied to the ASD population, which may capture or highlight other interesting computations necessary for adaptive mentalization. These kinds of approaches are likely to yield interesting quantitative characterizations of behavioral strategies and associated brain responses. Indeed, Yoshida and colleagues have reported interesting neural responses associated with parameters in their model in healthy volunteers [44] and have separately reported significant and interesting behavioral findings in a cohort of participants diagnosed with ASD [45]. In the latter account, the group revealed quantitative evidence for deficits in theory-of-mind processes, but also revealed heterogeneity in their ASD cohort. This kind of heterogeneity is to be expected in the broader ASD population and this finding is an important demonstration of the quantitative power that these games and applied computational approaches may have going forward.

These early applications of game theory to probe the behavioral space of social exchange have already revealed interesting aspects that may be critical for healthy social interaction. The advantages of these approaches will also be derived from subsequent investigations into the neural responses engaged during these games where scientists may further reduce the stimulus space to test the role of specific brain responses observed to be important. For example, Chiu and colleagues utilized the multi-round trust game to reveal reduced activity in the MCC during live social exchange that correlated with increased symptom severity on two social subscales and the overall score of the revised Autism Diagnostic Interview, Revised [26,46]. Lack of a neural signal for the first-person point-of-view in the cingulate cortex is suggested by the follow-up work presented here investigating neural responses to perspective taking in a experiment that controlled for effects of expertise familiarity (Figure 4). The results and current interpretation are consistent with a major subset of the clinical features of ASDs including deficits in imaginative play, communication, and social exchange. In addition, other literature suggests that the cingulate cortex may be a key anatomical region that differentiates humans and great apes from other primate species [55,56] and is a major site of cognitive integration serving many robust functions [57,58]. The MCC has specifically been implicated in the attribution of ownership of limbs and actions [47,59,60], willful production of speech and action [61,62], and more recently agent discrimination and perspective-taking during social exchange [46,47]. Our results suggest that the visual response in MCC demonstrated here may be related to an automatically generated representation akin to that elicited during first-person perspective-taking. The various roles suggested for the cingulate cortex imply that the function of this region is complex and may serve highly integrated and complex representations required for normal human cognition. Its role in mentalizing or theory-of-mind computations has yet to be investigated using a computationally rigorous model, suggesting an opportunity for future work in this domain.

Game theoretic approaches for measuring deviations from healthy decision-making behavior and the associated neural responses are a novel, but rapidly developing area in quantitative neurobiological approaches to understand mental disorders [30]. Neuroeconomic studies of healthy humans engaged in social decision-making tasks has revealed a number of interesting insights into brain responses associated with healthy human behavior and are beginning to make inroads to understanding ASDs. Early investigations into the possible genetic underpinnings have revealed that parameters associated with economic probes may show some significant heritability [63,64]. An advantage that the economic probes carry over traditional DSM criteria is in the objective and quantitative nature of their assessments of behavior. As these probes and computational descriptions of estimable model-based parameters develop so will the potential to quantify and decompose specific dimensions of social exchange. This is ultimately a task of reducing behavior and neural responses associated with mental illness to sub-components with the hope of providing refined targets for diagnostic specificity and novel (or improved) treatment strategies.

## Conclusions

The work presented here represents the earliest developments of the use of computational and game theoretic approaches to understand ASD. We present computationally obtained results from hyperscanned interactions between healthy participants and participants diagnosed with ASD. These interactions were social, however they were reduced to quantitative exchanges governed by the rules of the multi-round trust game. From these results a region of interest in the middle cingulate cortex was determined that was shown to differentiate self and other perspective-taking in an eyes-closed mental imagery task. Additionally we demonstrate that passively viewing pictures of oneself or another person elicits responses in this same region, which suggests the capacity to develop passive picture viewing assays for middle cingulate function using fMRI. Clustering algorithms applied to the behavioral gestures elicited during the multi-round trust game and Bayesian modeling approaches to social interaction were discussed to highlight the impact quantitative approaches to ASD are beginning to have. This work is clearly in its earliest stages, but is showing promise towards having objective and mathematically explicit theories of social cognition that hope to elucidate associated neurobiological mechanisms at multiple levels of investigation.

## Competing interests

The authors declare that they have no competing interests.

## Authors’ contributions

KTK analyzed and interpreted the human imaging data regarding the perspective-taking experiments in accomplished athletes and the self and other face viewing task and is a major contributor in the writing of this manuscript. JL assisted in the analysis of the athlete data. JS assisted in collecting the athlete data. PRM designed the structured imagery task, the multi-round trust task and analysis strategies for both interpretation and writing of this manuscript. All authors have read and approved this manuscript.

## Supplementary Material

Additional file 1 **Figure S1. **Structured Imagery Task. Click here for file

Additional file 2 **Figure S2. **Structured Imagery Task elicits self and other cingulate self-eigenmode responses. Click here for file
