# Supplementary figures and images for: New approaches to investigating social gestures in autism spectrum disorder
Source: J Neurodev Disord. 2012 May 24;4(1):14. doi: 10.1186/1866-1955-4-14 (PMC3436718; doi:10.1186/1866-1955-4-14)

*watch video*

*eyes-closed mental imagery*

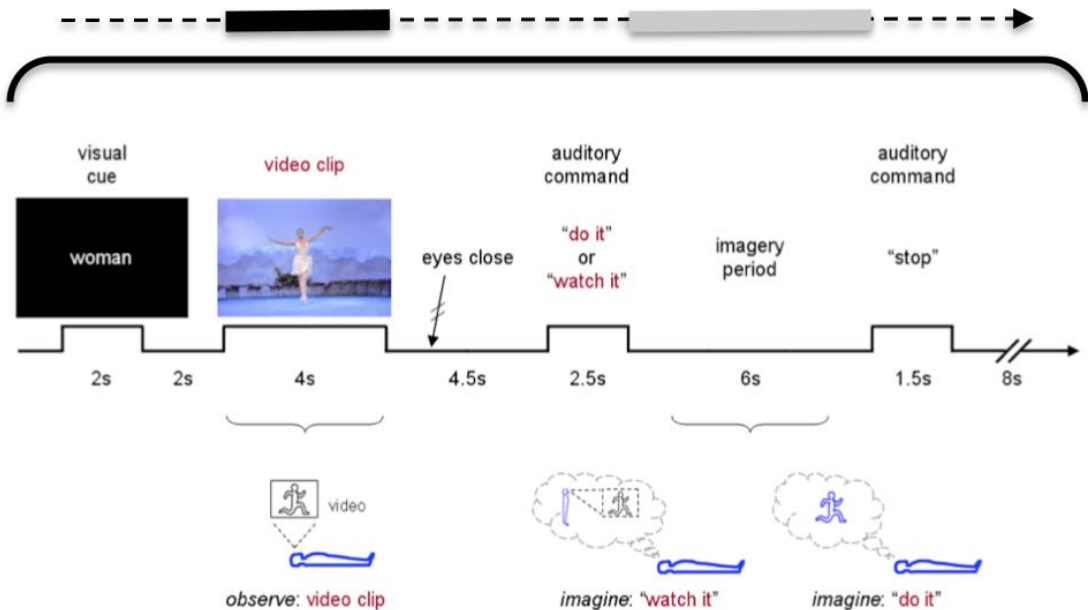

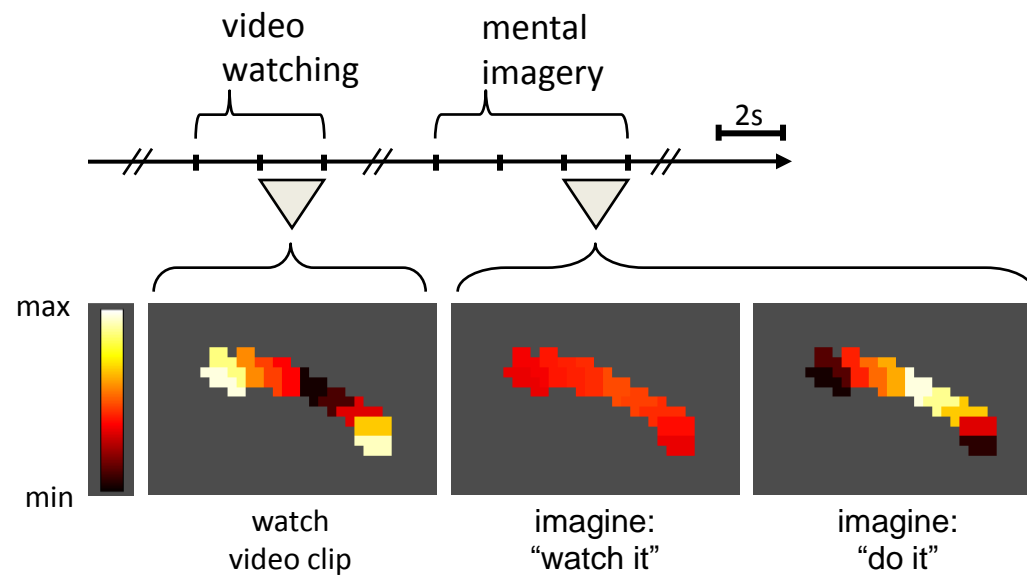

Supplement: Additional file 1 — Figure S1. Structured Imagery Task. [file 1866-1955-4-14-S1.pdf]
