# Supplementary material for: New approaches to investigating social gestures in autism spectrum disorder
Source: J Neurodev Disord. 2012 May 24;4(1):14. doi: 10.1186/1866-1955-4-14 (PMC3436718; doi:10.1186/1866-1955-4-14)
Supplement: Additional file 2 — Figure S2. Structured Imagery Task elicits self and other cingulate self-eigenmode responses. [file 1866-1955-4-14-S2.pdf]

## Supplemental Figures

**Supplementary Figure 1. Structured Imagery Task.** Accomplished-athletes (n = 81; Division-I Intercollegiate or Professional athletes from four different disciplines: ballet (n = 25), baseball (n = 14), football (n = 23), and soccer (n = 19) view video clips of sport-specific actions (taken from the respective athletic discipline represented in our subject pool) and subsequently perform eyes-closed motor-imagery or visual-imagery while being scanned using functional magnetic resonance imaging.

**Supplementary Figure 2. Structured Imagery Task elicits self and other cingulate self-eigenmode responses.** The mean BOLD response elicited during the respective phases of the Structured imagery task are projected onto the self-eigenmode. The magnitude of resulting projection coefficient is used to amplify the self-eigenmode basis function and plotted as a heat-map to demonstrate the contribution of the self-eigenmode in the respective phase of the task. Watching someone else perform an action (e.g. "watch video clip") elicits an "other" response (i.e., negative self-eigenmode); thinking about an action from the first-person point-of-view (e.g., **"**imagine: do it") elicits a "self" response (i.e., positive self-eigenmode); interestingly, thinking about the same actions, but from a removed third-person perspective (e.g., "imagine: watch it") elicits a neutralized self/other response (i.e., self-eigenmode is near zero).
